# A new species of *Lobellina* and first record of *Vietnura* from China (Collembola: Neanuridae: Neanurinae)

**DOI:** 10.3897/zookeys.807.24941

**Published:** 2018-12-17

**Authors:** Ji-Gang Jiang, Cheng-Wang Huang, Yun-Xia Luan

**Affiliations:** 1 College of Life and Environmental Sciences, Hunan University of Arts and Science, China Hunan University of Arts and Science Changde China; 2 Institute of Plant Physiology & Ecology, Shanghai Institutes for Biological Sciences, Chinese Academy of Sciences, Shanghai 200032, China Shanghai Institutes for Biological Sciences, Chinese Academy of Sciences Shanghai China; 3 Guangzhou Key Laboratory of Insect Development Regulation and Application Research, Institute of Insect Science and Technology & School of Life Science, South China Normal University, Guangzhou 510631, China South China Normal University Guangzhou China

**Keywords:** key, *Lobellinayinae* sp. n., new records, taxonomy

## Abstract

A new species of *Lobellina* Yosii, 1956 and a key to all species of the genus is provided. It is distinguished from all known members of the genus by its unique set of morphological characters: mandible with six teeth, cephalic chaeta O present, and free from tubercle Fr, cephalic tubercle Oc with three chaetae, cephalic tubercle Di separate, and tubercle Dl with four (sometimes three) chaetae, Ant. I with eight chaetae, and claw with an inner tooth. *Vietnuracaerulea* Deharveng & Bedos, 2000 is recorded from China for the first time. New localities of *Rambutanurahunanensis* Jiang & Dong, 2018 and *Vitronuraparaacuta* Wang, Wang & Jiang, 2016 from southwest China are also provided.

## Introduction

Maolan National Nature Reserve is located at Libo County, Qiannan Buyi and Miao Nationalities Autonomous Region of Guizhou Province, southwest China. It covers area of 212.85 km^2^ and is located in the subtropical monsoon humid climate zone. The main objectives of Maolan National Nature Reserve are the protection of the karst forest, and its rare animals and plants. It is from 430 to 1078 m above sea level. So far, no Neanuridae was reported from this reserve. During the field research at Maolan National Nature Reserve in 2015, four species of the subfamily Neanurinae were collected. They are described in the present paper.

## Materials and methods

Specimens were extracted from soil samples with the aid of Tullgren funnels or directly collected with an aspirator, and preserved in 95% ethanol. They were cleared in Nesbitt’s fluid and mounted on slides in Hoyer’s medium. Preparations were dried for 7–15 days in oven at 55 °C, then ringed with lacquer. The morphological characters were observed and figures were drawn using a phase contrast microscope Nikon 80i. Material is deposited in Shanghai Entomological Museum, Chinese Academy of Sciences.

The terminology and layout of the tables used in this paper follow Deharveng (1983), Deharveng and Weiner (1984), Smolis and Deharveng (2006), and Smolis (2008). The abbreviations used are listed below.

**General morphology**

Abd. abdomen

Ant. antenna

AOIII sensory organ of antennal segment III

Cx coxa

Fe Femur

Scx2 subcoxa 2

Ti tibiotarsus

Th. thorax

Tr trochanter

VT ventral tube

**Groups of chaetae**

Ag antegenital

An anal lobes

ap apical

ca centroapical

cm centromedial

cp centroposterior

d dorsal

Fu furcal

Vc ventrocentral

Veorve ventroexternal

Vea ventroexternoanterior

Vem ventroexternomedial

Vep ventroexteroposterior

Vel ventroexternolateral

Vec ventroexternocentral

Vei ventroexternointernal

Viorvi ventrointernal

Vl ventrolateral

**Tubercles**

An antennal

Fr frontal

Af antenno-frontal

Cl clypeal

De dorsoexternal

Di dorsointernal

Dl dorsolateral

L ateral

Oc ocular

So subocular

**Types of chaetae**

Ml long macrochaeta

Mc short macrochaeta

Mcc very short macrochaeta

me mesochaeta

mi microchaeta

ms s-microchaeta

s s-chaeta

bs s-chaeta on Ant. IV

miA microchaetae on Ant. IV

iv ordinary chaetae on ventral Ant. IV

or organite of Ant. IV

brs border s-chaeta on Ant. IV

i ordinary chaeta on Ant. IV

mou thin cylindrical chaetae on Ant. IV (“soies mousses”)

x labial sensory papilla

L’ ordinary lateral chaeta on Abd. V

## Taxonomy

### Tribe Lobellini Cassagnau, 1983

#### Genus *Lobellina* Yosii, 1956

##### Lobellina yinae sp. n.

Taxon classificationAnimaliaCollembolaNeanuridae

http://zoobank.org/FD11B2EC-7C4A-4480-BB09-BA7A3DEF144B

Figs 1 , 2 , 3 , 4–9 , Tables 1 , 2 , 3 , 4

###### Material.

**Holotype**, male, on slide. Maolan National Nature Reserve, Libo County, Guizhou Province, China. 25°16.400'N, 107°53.864'E, ca. 780 m above sea level, 22 July 2015. Collected by Cheng-Wang Huang, Yan Liang and Ai-Min Liu. **Paratype**, one subadult, same slide and data as holotype.

###### Etymology.

The species is named after Prof. Wen-Ying Yin, in honor of her important contributions to the study of Chinese soil animals.

###### Diagnosis.

Three pigmented eyes, mandible with six teeth, cephalic chaeta O present and free from tubercle Fr, cephalic tubercle Oc with three chaetae, cephalic tubercle Di separate, tubercle Dl with four (sometimes three) chaetae, Ant. I with eight chaetae, and claw with single inner tooth.

###### Description.

**Figure 1.**
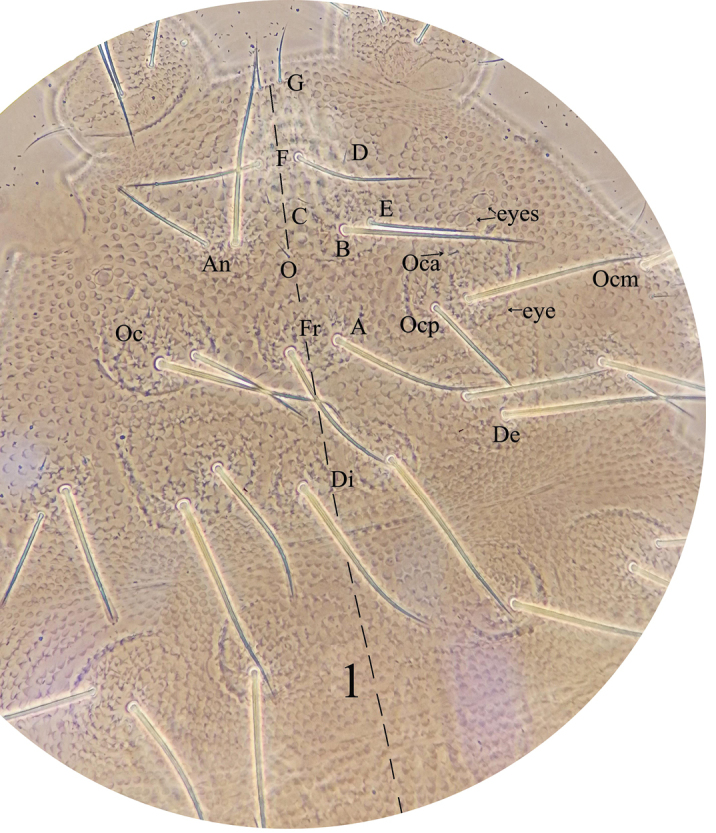
*Lobellinayinae* sp. n. dorsal tubercle and chaetotaxy of head.

**Figure 2.**
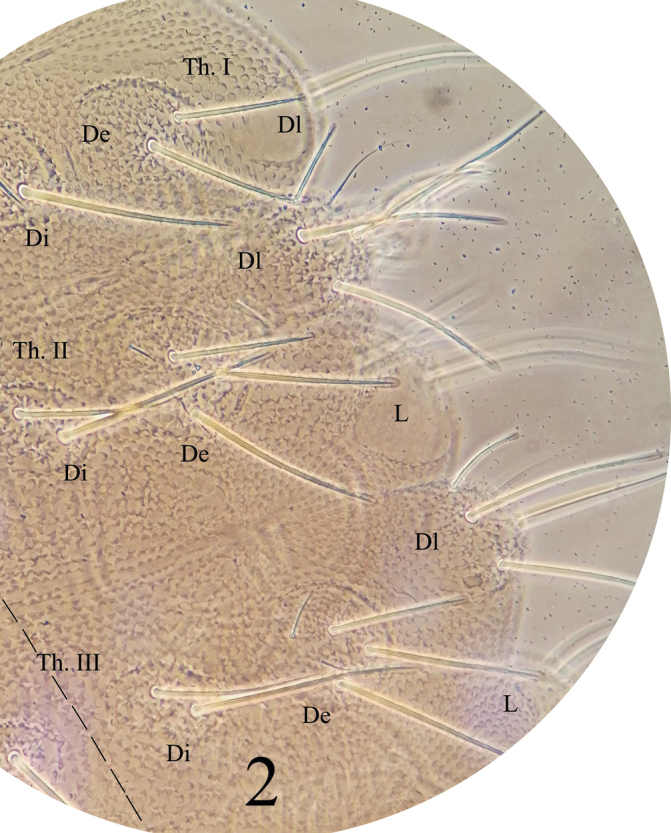
*Lobellinayinae* sp. n. dorsal tubercles and chaetotaxy on Th. I–III.

**Figure 3.**
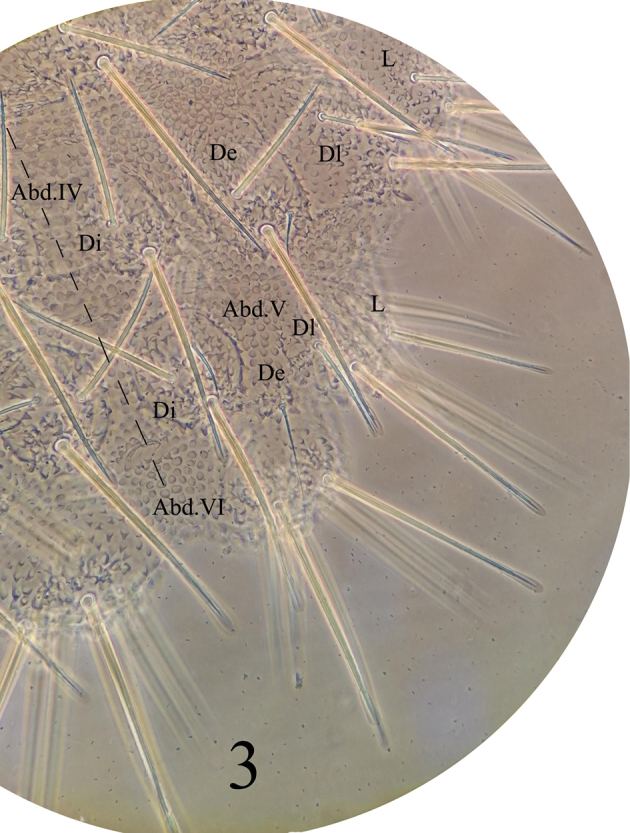
*Lobellinayinae* sp. n. dorsal tubercles and chaetotaxy on Abd. IV–VI.

**Figures 4–9.**
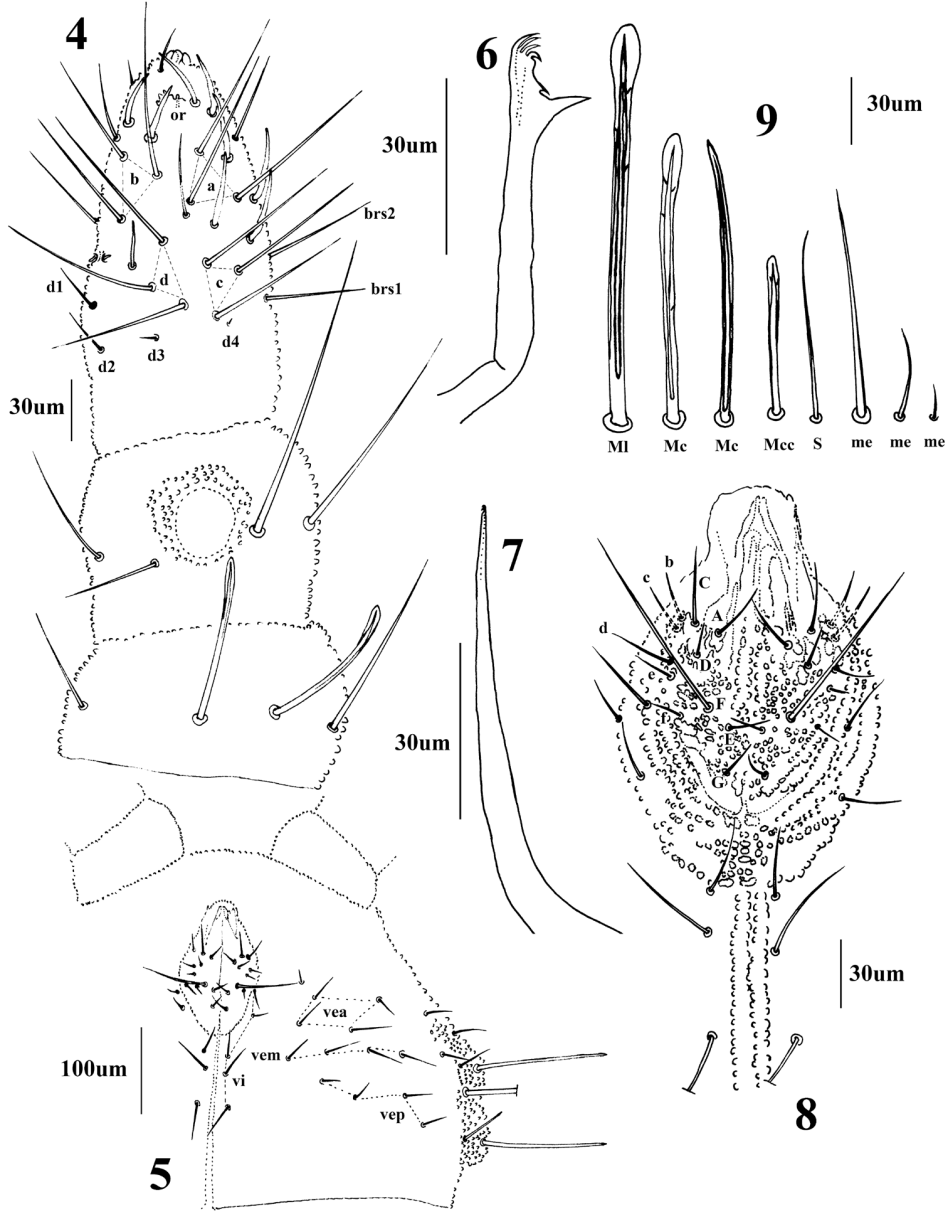
*Lobellinayinae* sp. n. **4** dorsal chaetotaxy of antenna **5** ventral chaetotaxy of head **6** mandible **7** maxilla **8** Labium **9** types of body chaetae.

*General* (Figs 1–3). Body length (without antenna) 1.8–2.1 mm. Cuticular granulations medium, tertiary granules absent, body without reticulations. Tubercles well developed on dorsal side of body. Body color red when alive, white in alcohol. Eyes 3+3, pigmented (Fig. 1). *Chaetal morphology* (Fig. 9). Dorsal ordinary chaetae of five types: Ml, Mc, Mcc, me, and mi. Macrochaetae Ml long, sheathed, weakly toothed and knobbed at apex. Macrochaetae Mc morphologically of two types: one is similar to Ml, but shorter, the other one with slightly pointed apex. Macrochaetae Mcc morphologically similar to Ml and shorter than Mc. Mesochaetae similar to ventral chaetae, thin, smooth, and pointed, with various length. Microchaetae shorter than mesochaetae, with acuminate tip. S-chaetae on terga thin, smooth, shorter than Mc, longer than Mcc. *Antenna* (Fig. 4 and Table 3). Antenna 4-segmented. Ant. I with eight chaetae. Ant. II with eleven chaetae and dorsally with a smooth circular area. Ant. III dorsally fused to Ant. IV. AOIII consists of two short rods, ventral ms and two longer sensory chaetae (sgd and sgv), sgd on the same level position of the two rods, each rod exposed in separate pit. Ant. IV dorsally with eight thickened and blunt sensilla, slender i-chaeta, and minute capitate organite (or). Apical bulb distinct, trilobed. Each of the eight sensilla distinctly differentiated, larger and two times shorter than “mou”-chaetae. Ventral chaetotaxy of Ant. III–IV is shown in Table 3, ap with eight bs and three miA, ca with two bs and two miA, cm with three bs and one miA, cp with six bs and seven miA. On ventral side of Ant. III, Vi, Vc, Ve respectively with four, four, five chaetae, Ant. III dorsally with 4–5 d chaetae, d1, d2, d3 as me, d4 as mi, d5 as mi and sometimes absent. *Mouthparts*. Buccal cone moderately long, labrum ventral sclerifications truncated (Fig. 8). Labrum chaetotaxy: 0/2, 2. Labium with normal chaetotaxy, and chaeta F almost three times as long as chaeta A, without papillae x (Fig. 8). Maxilla styliform, consisting of two fused lamellae, apically with two tiny teeth (Fig. 7). Mandible with four apical teeth, one middle tooth, and one large basal tooth (Fig. 6). *Dorsal chaetotaxy and tubercles of head* (Fig. 1 and Table 1). Head with 14 tubercles. Tubercle Cl with four chaetae: 2G+2F; tubercle An with four chaetae: B, C, D, E; tubercle Oc with three chaetae; tubercle Fr with three chaetae, chaeta O present, shifting between the two tubercles An; tubercle Di with a single chaeta; De with three chaetae; tubercle Dl separate from tubercle L+So, with four (or three) chaetae; tubercle L+So with 13 chaetae. *Dorsal chaetotaxy and tubercles of thorax* (Fig. 2 and Table 4). Thoracic dorsal tubercles complete. Th. I with three tubercles, tubercle Di with one chaeta; tubercle De with two chaetae; tubercle Dl with one chaeta. Th. II with four tubercles, tubercle Di with three chaetae; tubercle De with five chaetae (4+s); tubercle Dl with five chaetae and one ms (4+s+ms); tubercle L with three chaetae. Th. III with four tubercles, tubercle Di with three chaetae; tubercle De with five chaetae (4+s); tubercle Dl with five chaetae (4+s); tubercle L with three chaetae. *Dorsal chaetotaxy and tubercles of abdomen* (Fig. 3 and Table 4). Dorsum of Abd. I with four tubercles, tubercle Di with two chaetae; tubercle De with four chaetae (3+s); tubercle Dl with three chaetae; tubercle L with four chaetae. Tubercles and chaetae arrangements of Abd. II–III as on Abd. I. Abd. IV with four tubercles, tubercle Di with two chaetae; tubercle De with three chaetae (2+s); tubercle Dl with three chaetae; tubercle L with 5–7 chaetae. Abd. V with four tubercles, tubercle Di with three chaetae; tubercle De with one chaeta (s); tubercle Dl with four chaetae; tubercle L with seven chaetae (without s chaeta). Abd. VI bilobed, each side of Abd. VI with one tubercle, each tubercle with seven chaetae. No cryptopygy. S-chaetae formula on tergites as 0, 2+ms, 2/1, 1, 1, 1, 1. *Ventral chaetotaxy* (Fig. 5, Table 2). On ventral side of head, groups Vea, Vem, and Vep with five, four, four chaetae respectively. Group Vi on head with five chaetae. On Abd. I, VT with one proximal and three distal chaetae. On Abd. III, furca rudimentary with three chaetae, and without microchaeta. On Abd IV, group Vei, Vec, Vel respectively with one, two, four chaetae. On Abd. V, group Vl with 2–3 chaetae, Ag with 3–4 chaetae, chaeta L’ absent. Anal lobe with 14–15 chaetae and three mi. *Legs* (Table 4). Unguis with an inner tooth and without lateral tooth. Chaeta M on tibiotarsus present. Tibiotarsus of foreleg, midleg, and hindleg with 19, 19, 18 chaetae respectively.

**Table 1.** Cephalic dorsal tubercles and chaetotaxy of *Lobellinayinae* sp. n.

**Tubercle**	**Number of chaetae**	**Types of chaetae**	**Names of chaetae**
Cl	4	Ml	F
		me	G
An	4	M	B
		Mcc	E
		me	C, D
Fr	3	Ml	A
		me	O
Oc	3	Ml	Ocm
		Mcc	Ocp
		me or mi	Oca
Di	1	Ml	Di1
			Chaetal homology uncertain
De	3	Ml	De1
		Mc	De2
		mi	Di2
Dl	4 (3)	Mc+Mcc+2me (ormi)	Chaetal homology uncertain
L+So	13	4Ml+9me	Chaetal homology uncertain

**Table 2.** Cephalic ventral chaetotaxy of *Lobellinayinae* sp. n.

Group	Number of chaetae
Vi	5
Vea	5
Vem	4
Vep	4
Labium	11, 0 X

**Table 3.** Chaetotaxy of antenna of *Lobellinayinae* sp. n.

Segment, group	Number of chaetae	Segment, group	Number of chaetae
I	8	IV	or, 8 s, 12 mou, ? brs, 2 iv
II	11		
III	5 sensilla AOIII		
Ve	5	ap	8 bs, 3 miA
Vc	4	ca	2 bs, 2 miA
Vi	4	cm	3 bs, 1 miA
d	4(2me+2mi)–5(2me+3mi)	cp	1brs, 7 miA

**Table 4.** Postcephalic tubercles and chaetotaxy of *Lobellinayinae* sp. n.

**Terga**					**Legs**				
	Di	De	Dl	L	Scx2	Cx	Tr	Fe	T
Th. I	Ml	Ml+me	Ml	–	0	3	6	13	19
Th. II	Ml+Mc+mi	Ml+Mc+Mcc+me+ s	3Ml+Mcc+s+ms	Ml+2Mcc	2	7	6	12	19
Th. III	Ml+Mc+mi	Ml+Mc+Mcc+me+ s	3Ml+Mcc+s	Ml+2Mcc	2	8	6	11	18
**Terga**					**Sterna**				
Abd. I	Ml+Mc	Ml+Mc+ me+s	Ml+Mc+Mcc	Ml+Mc+2me	VT: 4				
Abd. II	Ml+Mc	Ml+Mc+me+s	Ml+Mc +Mcc	Ml+Mc+2me	Ve: 4–5, V1: 0				
Abd. III	Ml+Mc	Ml+Mc+me+s	Ml+Mc+Mcc	Ml+Mc+3me	Ve: 4, Fu: 3, 0 mi				
Abd. IV	Ml+Mc	Ml+Mc+s	Ml+Mc+Mcc	3Ml+2me or (3Ml+2me+2Mc)	Vei: 1, Vec: 1, Vel: 2 , Vl: 4				
Abd. V	Ml+Mc+me	s	2Ml+Mc+Mcc	7me or 1Mc+6me	Ag:3–4, Vl: 2–3, L’: 0				
Abd. VI	2Ml+5me				Ve: 14–15, An: 3 mi				

###### Ecology and distribution.

In fallen leaves of bamboo. *Lobellinayinae* sp. n. is only known from Libo (Fig. 16).

###### Remarks.

To date, 15 species of the genus *Lobellina* are known from Asia and one from Central America (Cuba) (Deharveng and Weiner 1984, Ma and Chen 2008, Smolis 2017, Jiang et al. 2018). The new species is similar to *L.montana* Deharveng & Weiner, 1984 and *L.paraminuta* Deharveng & Weiner, 1984 from Korea by the following characters: cephalic chaeta O free from tubercle Fr (shifting between two tubercles An), cephalic tubercle Dl separate from tubercle L+So, tubercle Oc with three chaetae, Abd. V with 3+3 dorsal tubercles and De separate from Dl, and claw with a distinct basal inner tooth. However, *L.yinae* sp. n. can be distinguished from *L.montana* and *L.paraminuta* by its mandible with six teeth versus seven, cephalic tubercle Dl with three or four chaetae versus five, tubercle De on Abd. I–III with four chaetae (3+s) versus three (2+s), and tubercle Dl on Abd. I–III with three chaetae versus two.

The new species is also similar to *L.fusa* Jiang, Wang & Xia, 2018 from China by the following characters: mandible with six teeth, maxilla styliform, tubercle Fr on head with three chaetae, tubercle Oc on head with three chaetae, Abd.V with 3+3 dorsal tubercles and De separate from Dl, and claw with a distinct basal inner tooth. However, the new species can be differentiated from *L.fusa* by the cephalic chaeta O of tubercle Fr free (not free in *L.fusa*), cephalic tubercles Di separated (fused in *L.fusa*), cephalic tubercle Dl with four chaetae (five in *L.fusa*), and each tubercle Dl on Abd. I–III with three chaetae (two chaetae in *L.fusa*).

#### Key to species of the genus *Lobellina* Yosii, 1956 (Modified from Jiang et al. 2018)

**Table:** 

1	Cephalic chaeta O present	**2**
–	Cephalic chaeta O absent	**7**
2	Chaeta O included in tubercle Fr	**3**
–	Chaeta O free on tubercle Fr	**4**
3	Body color yellow, mandible with seven teeth, tubercle Oc with 2 chaetae, ventral tube with 5+5 chaetae, cephalic tubercles Di separate	***L.nanjingensis* Ma & Chen, 2008 (China)**
–	Body color red, mandible with six teeth, tubercle Oc with three chaetae, ventral tube with 4+4 chaetae, cephalic tubercles Di fused	***L.fusa* Jiang, Wang & Xia, 2018 (China)**
4	Mandible with six teeth, Cephalic tubercle Dl with four (or three) chaetae	***L.yinae* sp. n. (China)**
–	Mandible with seven teeth, Cephalic tubercle Dl with five chaetae	**5**
5	Tubercle Dl on Th. II with six chaetae (4 +s+ms)	***L.montana* Deharveng & Weiner, 1984 (Korea)**
–	Tubercle Dl on Th. II with five chaetae (3+s+ms)	**6**
6	Tubercle Oc with mesochaeta Oca, Abd.V dorsally with 4+4 tubercles	***L.paraminuta* Deharveng & Weiner, 1984 (Korea)**
–	Tubercle Oc without chaeta Oca, Abd.V dorsally with 3+3 tubercles	***L.weinerae* Smolis, 2017 (Vietnam)**
7	Body macrochaetae smooth	**8**
–	Body macrochaetae serrate	**13**
8	Cephalic tubercle Oc with three chaetae	**9**
–	Cephalic tubercle Oc with two chaetae	**10**
9	Abd. V with 2+2 dorsal tubercles	***L.chosonica* Deharveng & Weiner, 1984 (Korea)**
–	Abd. V with 3+3 dorsal tubercles	***L.proxima* Deharveng & Weiner, 1984 (Korea)**
10	Tubercle Di on Abd. V with two chaetae	**11**
–	Tubercle Di on Abd. V with three chaetae	***L.minuta* (Lee, 1980) (Korea)**
11	Mandible with three teeth	***L.ipohensis* (Yosii, 1976) (Malaysia)**
–	Mandible with 6–8 teeth	**12**
12	Mandible with six teeth, tubercle De+Dl with six chaetae (5+s)	***L.pomorskii* Smolis, 2017 (Vietnam)**
–	Mandible with eight teeth, tubercle De+Dl with five chaetae (4+s)	***L.musangensis* (Yosii, 1976) (Malaysia)**
13	Cephalic tubercle Oc with two chaetae	**14**
–	Cephalic tubercle Oc with three chaetae	**15**
14	Abd. V with 2+2 dorsal tubercles	***L.ionescui* (Massoud & Gruia, 1974) (Cuba)**
–	Abd. V with 3+3 dorsal tubercles	***L.perfusionides* (Stach, 1965) (Vietnam)**
15	Abd. V with 2+2 dorsal tubercles	***L.roseola* (Yosii, 1954) (Japan)**
–	Abd. V with 3+3 dorsal tubercles	***L.kitazawai* (Yosii, 1969) (Japan)**

### Tribe Neanurini Börner, 1901 (sensu Cassagnau, 1983)

#### Genus Vietnura

Taxon classificationAnimaliaCollembolaNeanuridae

Deharveng & Bedos, 2000: new record to China

Vietnura caerulea Deharveng & Bedos, 2000: 209–214, figs 1–4 (Vietnam) new record to China

##### Material.

Two males on the same slide, one of them submature, 25°17.453'N, 107°56.359'E, elevation 880–900 m. Three individuals in alcohol, Coordinates: 25°17.516'N, 107°56.371'E, elevation 840 m. One specimen in alcohol, 25°17.483'N, 107°56.245'E, elevation 731 m. All of them were collected by Cheng-Wang Huang, Yan Liang & Ai-Min Liu, from Maolan National Nature Reserve, Libo County, Guizhou Province, China, on 19 July 2015. Material deposited in Shanghai Entomological Museum, Chinese Academy of Sciences.

##### Description of the Chinese specimens

**Figures 10–14.**
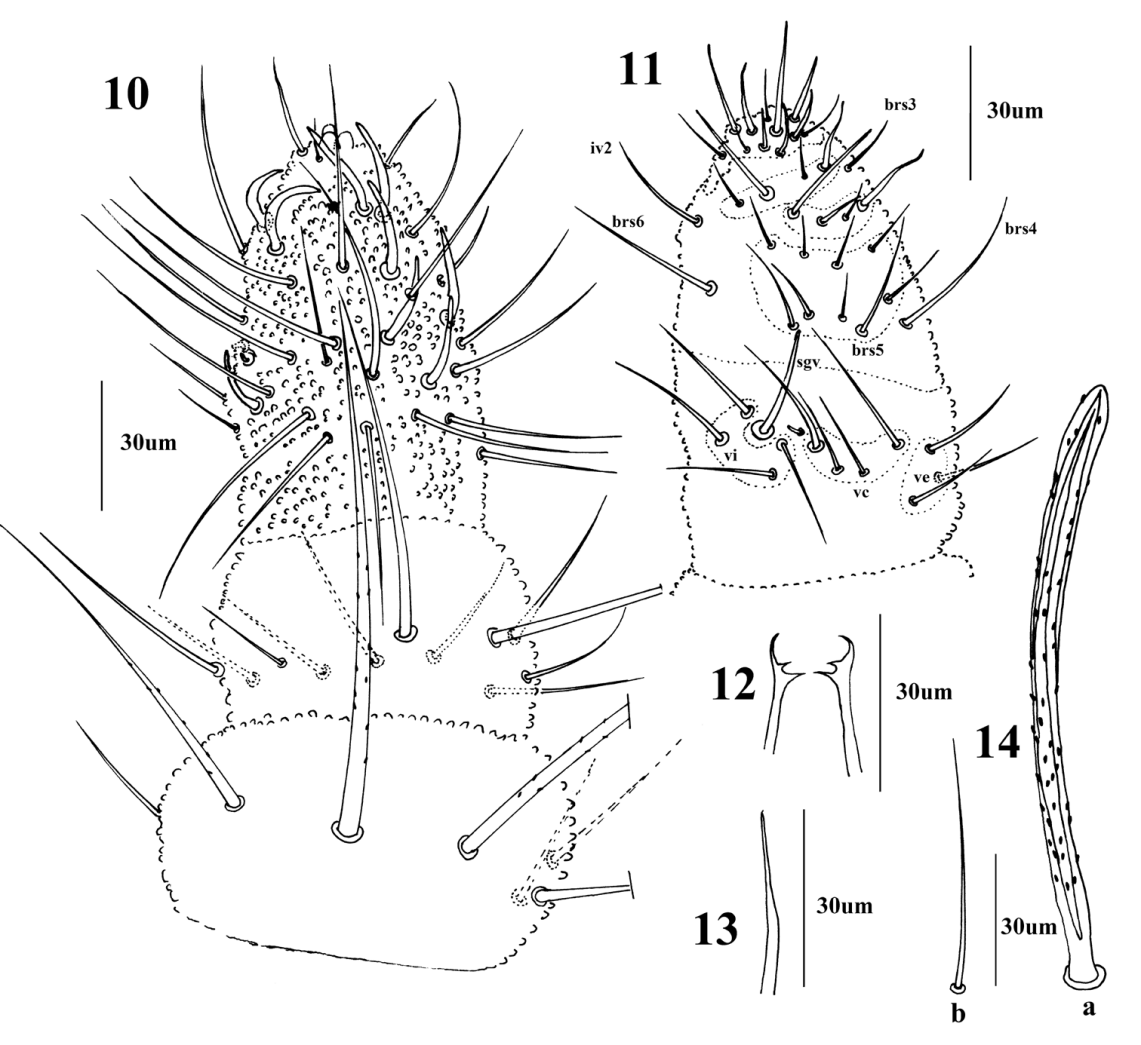
*Vietnuracaerulea* Deharveng & Bedos, 2000 **10** dorsal side of antenna **11** ventral side of Ant. III–IV **12** mandible **13** maxilla **14** body setae, a: macrochaeta, b: S-chaeta.

**Figure 15.**
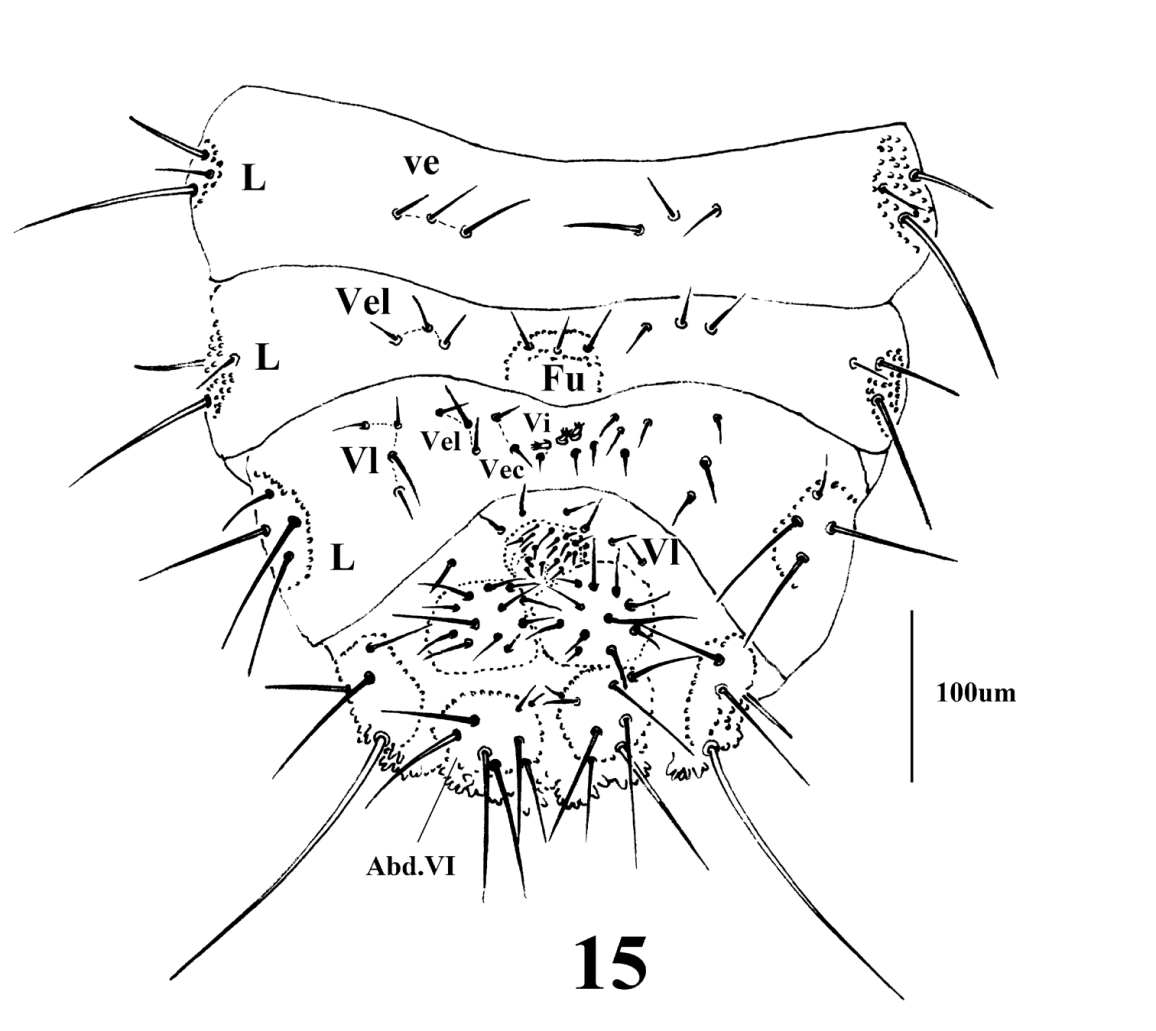
*Vietnuracaerulea* Deharveng & Bedos, 2000, ventral side of Abd. II–VI.

(Figs 10–15, Tables 5–7). *Body* length (without antenna) 0.9–1.1 mm. Cuticular granulations medium, tertiary granules developed, body with reticulations. Tubercles well developed on dorsal side of body. Body color blue alive and in alcohol. Eyes 2+2, small and pigmented, all on tubercles Oc. *Chaetal morphology* (Fig. 14). Dorsal ordinary chaetae of four types: Ml, Mc, Mcc, and me. Macrochaetae Ml long, sheathed, distinctly toothed and knobbed at apex (Fig. 14a). Macrochaetae Mc morphologically similar to long macrochaetae, but shorter. Macrochaetae Mcc morphologically similar to Mc and shorter than Mc. Mesochaetae similar to ventral chaetae, thin, smooth, and pointed, with various lengths. S-chaetae of tergites thin, smooth, shorter than Mc and slightly longer than Mcc “mou” (Fig. 14b). S-chaetae formula on tergites as 0, 2+ms, 2/1, 1, 1, 1, 1. *Antenna*. Antenna 4-segmented. Ant. I with seven chaetae. Ant. II with 10–11 chaetae. Ant. III dorsally fused to Ant. IV. AOIII consists of two short rods, one ventral msand two longer sensilla (sgd and sgv), sgd shifted basally to the back of the two rods, each rod exposed in separate pit (Fig. 10). Ant. IV dorsally with eight sensilla, slender i-chaeta, and minute capitate organite (or), apical bulb small, trilobed (Fig. 10). Sensilla thicker and shorter than “mou”-chaetae (Fig. 10). Ventral chaetotaxy of Ant. III–IV as in Fig. 11 and Table 6, group ap with seven bs and four miA, ca with two bs and two miA, cm with three bs and one miA, cp with eight miA and brs5. On ventral side of Ant. III, Vi, Vc, Ve respectively with four, four, three chaetae; dorsally with three d chaetae, d3 as mesochaeta (Fig. 10). Mouthparts. Buccal cone short, labral sclerifications not ogival. Labrum chaetotaxy: ?/2, 4. Labium with four basal, three distal, four lateral chaetae, without papillae x. Maxilla reduced, styliform (Fig. 13). Mandible reduced, tridentate (Fig. 12).

*Dorsal chaetotaxy and tubercles* (Table 7). Head with six tubercles. Tubercle Cl with four chaetae: two G and two F; tubercle Af+Oc with four chaetae: two B and two Ocm, chaeta O absent; tubercle Di+De with four chaetae: two Di1, two De1; tubercle Dl+L+So with eleven chaetae (5Ml+6me). Thorax and abdomen tubercles and chaetotaxy as in Table 7. Cryptopygy.

*Ventral chaetotaxy* (Fig. 15 and Table 5). On head, groups Vea, Vem and Vep with two, two, two chaetae respectively. Group Vi on head with five chaetae. VT with one proximal and three distal chaetae. On Abd. III, furca rudimentary with 3–4 chaetae, Vel with 3–4 chaetae. On Abd. IV, group Vei, Vec, Vel respectively with one, two, three chaetae, Vl with three or four chaetae. On Abd. V, group Vl with two chaetae, chaeta L’ absent, Ag with two chaetae. Anal lobe with twelve chaetae and one mi.

*Appendages*. Unguis without tooth. Chaeta M on tibiotarsus present. Tibiotarsus of foreleg, midleg and hindleg, respectively with 19, 19, 18 chaetae. Chaetotaxy of ventral tube and furcular remnant as in Table 7.

**Table 5.** Cephalic ventral chaetotaxy of *Vietnuracaerulea* Deharveng & Bedos, 2000.

Group	Number of chaetae
Vi	5
Vea	2
Vem	2
Vep	2
Labium	11, 0×

**Table 6.** Chaetotaxy of antenna of *Vietnuracaerulea* Deharveng & Bedos, 2000.

Segment, group	Number of chaetae	Segment, group	Number of chaetae
I	7	IV	or, 8 s, 12 mou, ? brs, 2 iv
II	10–11		
III	5 sensilla AOIII		
Ve	3	ap	7 bs, 4 miA
Vc	4	ca	2 bs, 2 miA
Vi	4	cm	3 bs, 1 miA
d	3	cp	1 brs, 8 miA

**Table 7.** Postcephalic tubercles and chaetotaxy of *Vietnuracaerulea* Deharveng & Bedos, 2000.

**Terga**					**Legs**				
	Di	De	Dl	L	Scx2	Cx	Tr	Fe	T
Th. I	Mc	Mc+Mcc	Mc	–	0	3	6	13	19
Th. II	Ml+Mcc	Mc+Mcc +s	Ml+2Mcc +s+ms	Ml+Mc+ Mcc	2	7	6	12	19
Th. III	Ml+MccorMl+2Mcc	Mc+Mcc +s	Ml+2Mcc +s	Ml+Mc+ Mcc	2	8	6	11	18
**Terga**					**Sterna**				
Abd. I	Ml+Mcc	Ml+Mcc +s	Ml+Mcc	Ml+Mcc+me	VT: 4				
Abd. II	Ml+Mcc	Ml+Mcc +s	Ml+Mcc	Ml+Mcc+me	Ve: 3				
Abd. III	Ml+Mcc	Ml+Mcc +s	Ml+Mcc	Ml+Mcc+me	Ve: 3–4, Fu: 3–4 me, mi: 0				
Abd. IV	Ml+Mcc	Ml+Mcc +s	Ml+Mcc	4me	Vei: 1, Vec: 2, Vel: 3, Vl: 3–4				
Abd. V	2(Ml+Mcc)*	Ml+Mcc+ 2me+s			Ag: 2, Vl: 2				
Abd. VI	7 (8)				Ve: 12, An: 1 mi				

*2 Di fused.

##### Ecology and distribution.

**Figure 16.**
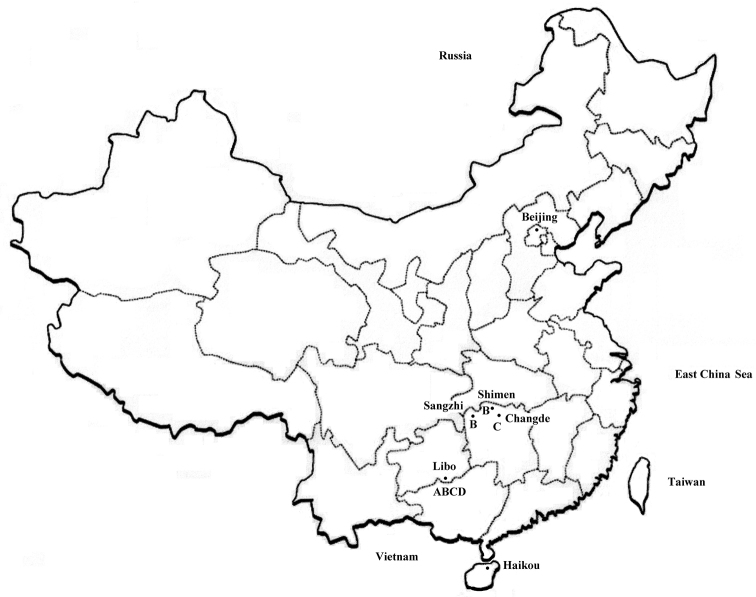
Map of China, with localities of *Lobellinayinae* sp. n. (**A**), *Rambutanurahunanensis* (**B**), *Vitronuraparaacuta* (**C**) and *Vietnuracaerulea* (**D**).

Among fallen leaves of bamboo and under broad-leaved trees in the forest. The species is described from Vietnam. In China, it is only known from Maolan National Nature Reserve, Libo County (Fig. 16).

##### Remarks.

*Vietnuracaerulea* is easily distinguished among Chinese Neanurinae by its blue body color, six tubercles on the head, 2+2 pigmented eyes on tubercle Af+Oc, and reduced mandible and maxilla. Additionally, Ve chaetal group of Abd. IV has 3–5 shortened, thickened, and distally ciliated chaetae (male), claw is toothless, and hypotrichosis is developed on body tubercles.

### Tribe Paleonurini Cassagnau, 1989

#### Genus *Rambutanura* Deharveng, 1988

##### Rambutanura hunanensis

Taxon classificationAnimaliaCollembolaNeanuridae

Jiang & Dong, 2018

Rambutanura hunanensis Jiang & Dong, 2018: 377–386, figs 1 –14 (China)

###### Material.

One juvenile, body length 2.2 mm, on slide; two specimens in alcohol, probably juvenile. Maolan National Nature Reserve, Libo County, Guizhou Province, China, 25°16.400'N, 107°53.864'E, ca. 890 m above sea level. 19 July 2015. Collected by Cheng-Wang Huang, Yan Liang, and Ai-Min Liu.

###### Diagnosis.

The specimen from Libo County is characterized by its body without long digitate tubercles and tertiary granules, 2+2 depigmented eyes, mandible with four teeth, maxilla styliform, head with eight tubercles (Cl, Af, 2 Oc, 2 Di+De, 2 Dl+L+So), claw with a big inner tooth, and ventral tube with 5–6 chaetae. These characters are similar to those of *Rambutanurahunanensis* Jiang & Dong, 2018 from Hunan Province; however, the presence of only four chaetae on genital plate reveals the immaturity of the Maolan specimens.

###### Remarks.

The distribution of *R.hunanensis* is given in Fig. 16. The species has been collected from other localities in China, such as Huping Mountain, Shimen County, Hunan Province (unpublished). It is probably widely distributed in central and southwest China.

#### Genus *Vitronura* Yosii, 1969

##### Vitronura paraacuta

Taxon classificationAnimaliaCollembolaNeanuridae

Wang, Wang & Jiang, 2016

Vitronura paraacuta Wang, Wang & Jiang, 2016: 183–196, figs 1–7 (China)

###### Material.

Two females, submature, on slides, five specimens in alcohol, Maolan National Nature Reserve, Libo County, Guizhou Province, China, 25°16.400'N, 107°53.864'E, ca. 880 m above sea level. 19 July 2015. Collected by Cheng-Wang Huang, Yan Liang, and Ai-Min Liu.

###### Diagnosis.

The characters of the specimens from Maolan are consistent with those of *Vitronuraparaacuta* Wang, Wang & Jiang, 2016: body tubercles well differentiated, head with 14 tubercles (only cephalic tubercle L fused to So), 2+2 depigmented eyes, mandible with four teeth, maxilla styliform, tubercles Fr and Oc with three chaetae each, and claw with an inner tooth.

###### Remarks.

The arrangement of the dorsal body tubercles and numbers of chaetae on dorsal tubercles of *V.paraacuta* are very similar to those of *V.dentata* Deharveng & Weiner, 1984 from Korea. However, *V.paraacuta* can be differentiated from *V.dentata* by almost smooth body macrochaetae, four teeth on mandible, chaetae Di2, De2 on cephalic tubercle De and chaeta Oca on cephalic tubercle Oc being mesochaetae (*vs* serrated body macrochaetae, three teeth on mandible, chaetae Di2, De2 on cephalic tubercle De and chaeta Oca on cephalic tubercle Oc being microchaetae in *V.dentata*). The distribution of *V.paraacuta* is given in Fig. 16.

## Supplementary Material

XML Treatment for Lobellina yinae

XML Treatment for Vietnura

XML Treatment for Rambutanura hunanensis

XML Treatment for Vitronura paraacuta
